# Origins of Hyperbolicity in Color Perception

**DOI:** 10.3390/jimaging6060042

**Published:** 2020-06-04

**Authors:** Nicoletta Prencipe, Valérie Garcin, Edoardo Provenzi

**Affiliations:** 1 Institute of Mathematics, Université de Bordeaux, CNRS, Bordeaux INP, IMB, UMR 5251, F-33400 Talence, France or nicoletta.prencipe1@huawei.com (N.P.); edoardo.provenzi@math.u-bordeaux.fr (E.P.); 2 Huawei Technologies France SASU, 06410 Sophia Antipolis, France

**Keywords:** space of perceived colors, Yilmaz’s model, Lorentz transformations, hyperbolic geometry

## Abstract

In 1962, H. Yilmaz published a very original paper in which he showed the striking analogy between Lorentz transformations and the effect of illuminant changes on color perception. As a consequence, he argued that a perceived color space endowed with the Minkowski metric is a good approximation to model color vision. The contribution of this paper is twofold: firstly, we provide a mathematical formalization of Yilmaz’s argument about the relationship between Lorentz transformations and the perceptual effect of illuminant changes. Secondly, we show that, within Yilmaz’s model, the color space can be coherently endowed with the Minkowski metric only by imposing the Euclidean metric on the hue-chroma plane. This fact motivates the need of further investigation about both the proper definition and interrelationship among the color coordinates and also the geometry and metrics of perceptual color spaces.

## 1. Introduction

Yilmaz’s paper [1] belongs to a surprisingly rich list of contributions to color theory by theoretical physicists. One of the founding fathers of quantum mechanics, E. Schrödinger is among the most famous, with his benchmark axiomatic work on color perception [2]. In more recent years, also S. Weinberg [3] and A. Ashtekar [4], to quote but two, wrote papers about color. The common denominator of all these contributions is the geometry of the perceived colors space C. The geometric models of gravitation and fundamental interactions play a central role in theoretical physics, thus it is not surprising that these scientists were interested in the analysis of the geometry of C.

As we will detail in this paper, H. Yilmaz had the brilliant idea to point out the analogy between perceived colors theory and the special theory of relativity by observing that the saturation of spectral colors is not only an upper bound, but also a perceptually invariant feature with respect to the (broadband) illuminant used to light up a visual scene. The analogy between this property of color saturation and the constancy of the speed of light in vacuum, when measured by inertial observers, in special relativity is clear.

Yilmaz determined, on the base of three results that he claimed coming from experiments, a law for the perceptual effect on color perception induced by a change of illuminant. This law turns out to be the direct analogous of Lorentz transformations. From this, he argued that a color space endowed with a 3D Minkowski metric could be a valid alternative to the classical CIE colorimetric spaces, which are equipped with a Euclidean metric to measure perceived color distances.

The purpose of this paper is double: on one side, we formalize some aspects of Yilmaz’s model that we deem not mathematically clear and, on the other side, we complete its work by showing that it is possible to coherently endow C with the Minkowski metric only if we assume the hue-chroma plane to be endowed with the Euclidean metric.

The structure of the paper is as follows: in Section 2 we start by recalling Yilmaz’s experiments and the results on which his model is based, then, in Section 3, we recast his description in a clearer mathematical framework and we point out the critical issues of the original model. In Section 4 the analogy with special relativity, Lorentz transformations and the Minkowski metric is discussed in detail. In Section 5 the most ambiguous result claimed by Yilmaz, the one referred as experiment 3, is shown to be linked to the relativistic aberration effect. Finally, in Section 6, we discuss our results together with some ideas for future research perspectives.

## 2. Yilmaz’s Experiments

We are going to introduce the experiments on which Yilmaz based his model. For the sake of clarity, we first introduce the notation and nomenclature used in the rest of the paper.

### 2.1. Notation and Nomenclature for Yilmaz’s Model

To develop his model, Yilmaz considered trichromatic observers and the color space C embedded in the closed upper half-space in the Euclidean 3D space, i.e., $H:=\left\{\left(\alpha ,\beta ,\gamma \right)|\left(\alpha ,\beta \right)\in R^{2},\gamma \geq 0\right\}≅R^{2}\times R_{0}^{+}$. (α,β,γ) are the components of a color F∈C with respect to a basis $\left(\hat{\alpha },\hat{\beta },\hat{\gamma }\right)$ of $R^{3}$, (α,β) are called chromaticity coordinates and γ is the achromatic one, called *lightness* in [1]. In this section, we use the word ‘color’ without specifying in which context the color is perceived. We will formalize this concept in Section 3.

The polar coordinates in the so-called hue-chroma plane are (ϕ,ρ), where α=ρcosϕ and β=ρsinϕ, ϕ being associated to the *hue* and ρ to the *chroma* of F∈C. The values of ϕ and ρ/γ are provided by psycho-visual experiments. This implies that the values of α and β are defined modulo the value of γ. In fact, in the rest of the paper γ will be treated as an unknown parameter which will intervene only in intermediate steps of the computations and not in the final results.

**Figure 1:**
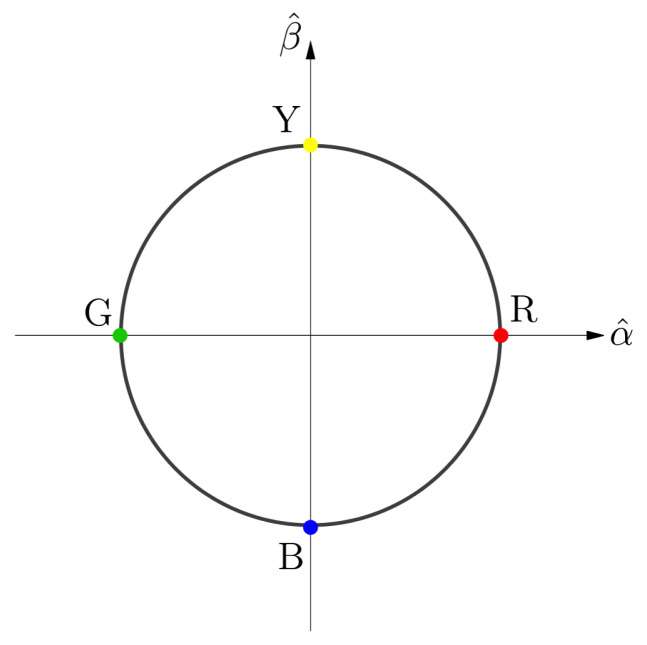
The (α,β)-plane.

It is customary, although arbitrary, to identify the hue corresponding to particular values of ϕ with the following standard hues: ϕ=0 is red *R*, ϕ=π/2 is yellow *Y*, ϕ=π is green *G* and ϕ=3π/2 is blue *B*. Coherently with this identification, from now on, as shown in Figure 1, the $\hat{\alpha }$ axis will be identified with the R−G direction and the $\hat{\beta }$ axis with the Y−B direction.

Following the standard colorimetric definition, Yilmaz relates color *saturation* σ with ρ and γ via the following equation:

(1) σ=ρ/γ.

We recall that Maxwell’s plane is the normal plane to the achromatic axis with value of γ fixed to 1, in this plane, ρ and σ can be identified and they represent the radial distance from the γ axis.

The half-line defined by γ≥0, ρ=0, is called *achromatic axis*, the maximum perceivable value for γ is denoted with $\gamma_{\max}$. For all the values $\gamma >\gamma_{\max}$ cone receptors are saturated due to *glare*. The origin *O* corresponds to the sensation of black.

It is a known fact gathered by psychophysical experiments that the saturation of spectral colors, i.e., narrow-band lights, is maximal. Yilmaz denoted with $\Sigma_{\phi }$ the maximal saturation sensation induced by a narrow-band light perceived with a hue ϕ. In spite of the fact that we will eventually drop the dependency of Σ on ϕ, for the moment we will be faithful to Yilmaz’s notation.

**Figure 2:**
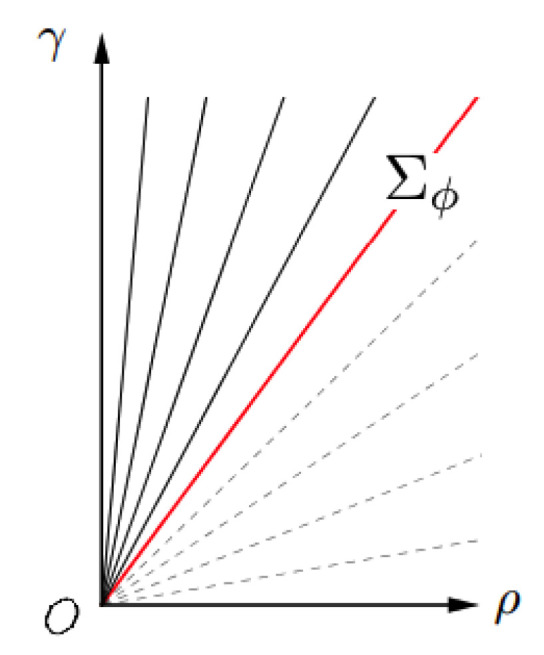
The existence of a minimal slope 1/Σ in the quadrant ϕ= constant.

Figure 2 shows the quadrant defined by ϕ= constant. The existence of a maximal saturation $\Sigma_{\phi }$ clearly implies that the slope 1/σ has a lower bound given by $1/\Sigma_{\phi }$.

### 2.2. Yilmaz’s Experiments

**Figure 3:**
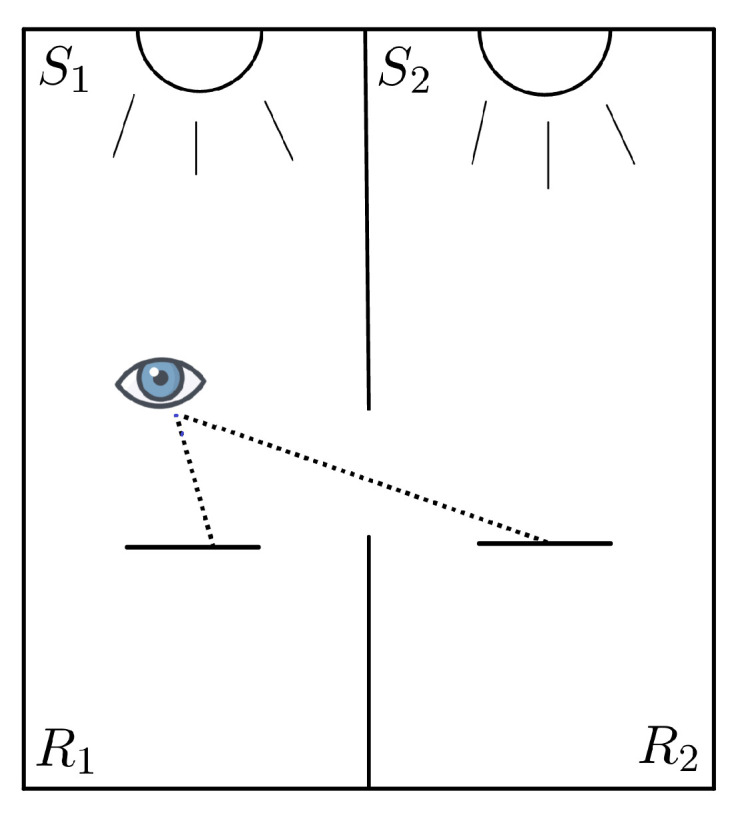
The experimental apparatus considered by Yilmaz.

The generic apparatus for the experiments described by Yilmaz in [5] is shown in Figure 3, where we can see two identical rooms $R_{1}$ and $R_{2}$, separated by a common wall with a thin hole and illuminated by the sources of light $S_{1}$ and $S_{2}$. Both rooms are painted with a non-selective Lambertian white paint. A piece of white paper is divided in two parts and they each one is placed in one of the rooms, so that an observer can perceive them simultaneously. The key point is that one piece is seen directly and the other through the hole.

The illumination $S_{1}$ of room $R_{1}$ will always be provided by near-daylight broadband illuminants. Instead, the illumination of room $R_{2}$ will be provided by a light source $S_{2}$ that can also be narrow-band. The perceived colors are compared with the help of a set of Munsell chips enlighted by the same illuminant under which the observer is adapted.

#### 2.2.1. The First Experiment

In this first experiment, the sources $S_{1}$ and $S_{2}$ are chosen to be two different broadband illuminants of near-daylight chromaticity, *I* and $I^{\prime }$, respectively. An observer placed in $R_{i}$ will adapt to $S_{i}$ and the piece of paper placed in $R_{i}$ will be perceived as white, i=1,2. However, Yilmaz noticed that, if an observer, placed in one of the two rooms, looks at the piece of paper in the adjacent room through the thin hole, then it will appear with a certain hue ϕ and saturation σ. By switching the rooms, the piece of paper in the adjacent room will be matched with a Munsell chip of opposite hue, i.e., π+ϕ, but with approximately the same saturation σ. To fix the ideas we choose the hues ϕ and π+ϕ to be red and green, respectively. We consider this description of the first experiment more correct than the one performed by Yilmaz, which used the symbol −σ to denote the perceived saturation of the opponent hue. In fact, saturation has been defined as a non-negative quantity, so the use of −σ is meaningless.

This experiment is extremely interesting because the thin hole in the wall is a trick that permits to show how an observer’s reference for white changes when the illumination varies. The immediate consequence is that *color perception is a relative phenomenon*, which depends on the illuminant to which the observer is adapted.

It is worth underlining that this experiment must be performed in such a way that *local retinal adaptation* is prevented in the area covered by the thin hole. This can be done, for example, by allowing only a limited time aperture of the thin hole with the help of a suitable time-dependent shutter.

#### 2.2.2. The Second Experiment

In the second experiment Yilmaz chooses $S_{2}$ to be a narrow-band source with a spike in the red region of the visual spectrum. Yilmaz reported that, if an observer in $R_{1}$ is adapted to the broadband near-daylight illuminant *I* and looks at the piece of paper in $R_{2}$, he/she will perceive it as having same hue ϕ=0 and with maximal saturation $\Sigma_{R}$. If we change the illuminant *I* with the illuminant $I^{\prime }$ used in the first experiment and we wait for the adaptation of the observer in $R_{1}$ to the new illuminant, then the piece of paper in $R_{2}$, seen through the hole, will still be perceived as having same hue ϕ=0 and maximal saturation $\Sigma_{R}$.

Yilmaz justifies experimentally this claim by saying that, in both cases, the perceived saturation is reported to be too high to be replicated by any of the Munsell chips, i.e., the observer is able to identify the perceived hue as red, but all of the Munsell chips have saturation strictly smaller than the perceived one.

From the first experiment, we know that the change of perceived hue caused by the transformation from *I* to $I^{\prime }$ acts on the red-green axis.

The particular choice of the red-green axis seems to be the only one really tested by Yilmaz, however, theoretically, nothing prevents to choose any other chromatic opponent axis.

#### 2.2.3. The Third Experiment

This final experiment is similar to the second one, but with a crucial difference. Here $S_{2}$ is chosen to be a narrow-band source of light with spike in the yellow part of the spectrum, i.e., orthogonal to the R−G axis. The observer is always placed in $R_{1}$. When $S_{1}$ is equal to *I* he/she perceives $S_{2}$ through the hole with yellow hue, i.e, ϕ=π/2, and with a saturation which is, again, too high to be found among the set of Munsell chips and then it is set to $\Sigma_{Y}$. When the illuminant $S_{1}$ changes from *I* to $I^{\prime }$, no variation in saturation is reported, it is still maximal and equal to $\Sigma_{Y}$, but the hue perception of the piece of paper in $R_{2}$ seen from the hole changes by an amount φ such that

(2) sinφ≃σ/Σ.

At page 12 of [5], Yilmaz writes: ‘[…] these conclusions based on experiment are […] only approximate […]’, from this we understand that experiments have in fact been performed and data have been gathered. However, it is also clear that such a precise formula as Equation (2) to determine the hue shift φ is, at least, doubtful. We will turn back on this issue in Section 3.4.

The aim of this paper is to mathematically analyze Yilmaz’s model and its consequences, for this reason, in spite of this debated issue, we will consider this data as rigorous. However, it is clear that further psychophysical experiments would be extremely valuable to confirm or confute Equation (2) and the other data reported by Yilmaz.

## 3. Recasting Yilmaz’s Model in a Mathematical Framework

In section IV ‘Transformation formulae’ of his paper [5], Yilmaz’s looked for *a transformation from the coordinates of a color described by an observer adapted to a broadband illuminant I to those of an observer adapted to different broadband illuminant $I^{\prime }$*. He deduced, from the three experiments previously discussed, what he claimed to be a linear approximation of this transformation. Such a transformation leaves of course the black point *O* fixed.

Unfortunately, the mathematical exposition of Yilmaz lacks of rigor. Our contribution is this section is to introduce a precise notation and a suitable language that will allow us to translate into a rigorous and coherent mathematical framework the information provided by Yilmaz.

We start with the definition of visual stimuli.

- If Λ⊂R is the compact subset of R containing the visible wavelengths, typically Λ=[380,780] nm, then a *visible light* can be identified with a finite energy non-negative function defined on Λ, i.e., an element of the space $L_{+}^{2}\left(\Lambda \right)=\{f:\Lambda \to R^{+}\;:\;\int_{\Lambda }{\left|f\right|}^{2}<+\infty \}$;
- We call *visual stimulus* in Yilmaz’s experiment the spectrum of visible light reflected by either a piece of white paper, or a Munsell chip illuminated by a visible light representing an illuminant entering the eye of an observer. More precisely, the piece of white paper will be illuminated by both broadband and narrow-band illuminants, while the Munsell chips will only be illuminated by broadband illuminants;
- $F,F^{\prime }$ will denote a visual stimulus provided by the visible light reflected by an object enlighted by the illuminant *I* or $I^{\prime }$, respectively. The object surface can be either the piece of white paper, and in that case we will write $W,W^{\prime }$, or a Munsell chip;
- $\tilde{R},\tilde{Y}$ will indicate the visual stimulus provided by the piece of white paper illuminated by the narrow-band illuminants with spike in the red or yellow region, respectively.

Yilmaz assumed that an observer adapted to a broadband illuminant, *I* or $I^{\prime }$, analyzing colors by the comparison with a set of Munsell chips enlighted by *the same* illuminant, defines a vector basis of $R^{3}$, $B=\{\hat{\alpha },\hat{\beta },\hat{\gamma }\}$ or $B^{\prime }=\left\{{\hat{\alpha }}^{\prime },{\hat{\beta }}^{\prime },{\hat{\gamma }}^{\prime }\right\}$, respectively. Thus, the use of B and $B^{\prime }$ will be always implicitly correlated with a triple given by an illuminant, an observer adapted to it and a set of Munsell chips used for color comparison. If even a single one of these element is lacking, then the color identification process in Yilmaz’s model is not possible.

**Figure 4:**
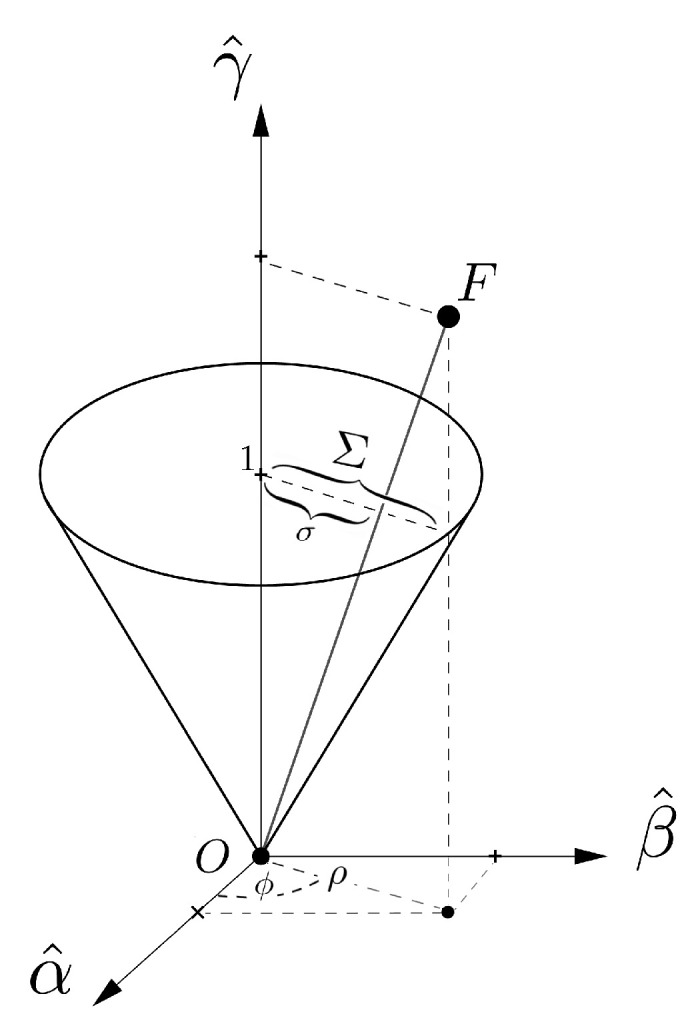
The color cone C for an ideal observer, no glare, nor visibility threshold is considered in this representation.

This observation explains why we will consider the effective available space for perceived colors in Yilmaz’s setting as the volume contained in the *open cone* shown in Figure 4, denoted by C⊂H and described by the equation:

(3) $$C=\{F=\left(\phi ,\rho ,\gamma \right)\in H\;|\;\rho <\Sigma_{\phi }\gamma \}.$$

We assume $\Sigma_{\phi }$ to be the same for all observers adapted to any illuminant. Since it is impossible for an observer to replicate with a Munsell chip the maximal saturation $\Sigma_{\phi }$ of a narrow band visual stimulus, we consider the color sensation produced by such a visible light as a point belonging to the surface $\partial C=\{F=\left(\phi ,\rho ,\gamma \right)\in H\;|\;\rho =\Sigma_{\phi }\gamma \}$ of the cone previously defined.

The symbols B and $B^{\prime }$ will be used as a subscript for the visual stimuli to indicate the illuminant to which the observer is adapted: B for *I* and $B^{\prime }$ for $I^{\prime }$. Note that the basis subscript is extremely important because it underlines the central role of the observer, i.e., the basis with respect to which the coordinates are written. Without an observer a “perceived color" is just a “stimulus”, in the same way as a point of a vector space is just an abstract (coordinate-free) concept without a basis which describes it in terms of coordinates.

Yilmaz considers the change of basis from B to $B^{\prime }$ to be the *linear approximation* (actually, as we will prove later, this is not an approximation, since the transformation is indeed linear) of the illuminant transformation from *I* to $I^{\prime }$ and denotes the associated matrix as $\Omega \equiv \Omega_{II^{\prime }}\in GL\left(3,R\right)$ (we recall that GL(n,R) indicates the so-called general linear group of degree *n* over R, which is the set of all the invertible matrices n×n with entries in R). Ω is naturally required to be invertible because we can reverse the transformation by switching from one illuminant adaptation to the other, i.e., $\Omega^{-1}=\Omega_{II^{\prime }}^{-1}=\Omega_{I^{\prime }I}$.

In order to determine the coefficients of Ω, Yilmaz considered the following equations:

$$\left\{\begin{array}{c} \Omega W_{B}=W_{B^{\prime }}\\ \Omega^{-1}W_{B^{\prime }}^{\prime }=W_{B}^{\prime } \end{array}\right.\;\mathrm{and}\;\;\left\{\begin{array}{c} \Omega {\tilde{R}}_{B}={\tilde{R}}_{B^{\prime }}\\ \Omega {\tilde{Y}}_{B}={\tilde{Y}}_{B^{\prime }}. \end{array}\right.$$

These equations are the translation of the three Yilmaz experiments in our notation.

If we denote with ${\left(\alpha ,\beta ,\gamma \right)}^{t}$ or ${\left(\alpha^{\prime },\beta^{\prime },\gamma^{\prime }\right)}^{t}$ the coordinates of a color perceived by an observer adapted to *I* or $I^{\prime }$, respectively, then

(4) $$\left(\begin{array}{c} \alpha^{\prime }\\ \beta^{\prime }\\ \gamma^{\prime } \end{array}\right)=\left(\begin{array}{ccc} \Omega_{11} & \Omega_{12} & \Omega_{13}\\ \Omega_{21} & \Omega_{22} & \Omega_{23}\\ \Omega_{31} & \Omega_{32} & \Omega_{33} \end{array}\right)\left(\begin{array}{c} \alpha \\ \beta \\ \gamma \end{array}\right)\;\Leftrightarrow \;\left\{\begin{array}{c} \alpha^{\prime }=\Omega_{11}\alpha +\Omega_{12}\beta +\Omega_{13}\gamma \\ \beta^{\prime }=\Omega_{21}\alpha +\Omega_{22}\beta +\Omega_{23}\gamma \;.\\ \gamma^{\prime }=\Omega_{31}\alpha +\Omega_{32}\beta +\Omega_{33}\gamma \end{array}\right.$$

At page 14 of [5], Yilmaz analyzes, among all possible illuminant changes, the situation in which the couple *I* and $I^{\prime }$ produces a color coordinate transformation only along the α-axis, i.e., the R−G direction, leaving the β-axis totally unaffected, i.e., $\beta^{\prime }=\beta$. For this to happen, the second equation of (4) tells us that we must have $\Omega_{21}=\Omega_{23}=0$ and $\Omega_{22}=1$.

The preservation of the β-axis for the inverse transformation, represented by $\Omega^{-1}{\left(\alpha^{\prime },\beta^{\prime },\gamma^{\prime }\right)}^{t}={\left(\alpha ,\beta ,\gamma \right)}^{t}$, can be verified with a straightforward computation to imply that $\Omega_{12}=\Omega_{32}=0$.

So, the matrix Ω has the following form

(5) $$\Omega =\left(\begin{array}{ccc} \Omega_{11} & 0 & \Omega_{13}\\ 0 & 1 & 0\\ \Omega_{31} & 0 & \Omega_{33} \end{array}\right).$$

The remaining coefficients, i.e., $\Omega_{ij}$, i,j=1,3, will be determined by translating into formulae the three Yilmaz’s experiments.

### 3.1. Coefficients from the First Experiment: The White Point Transformation

**Figure 5:**
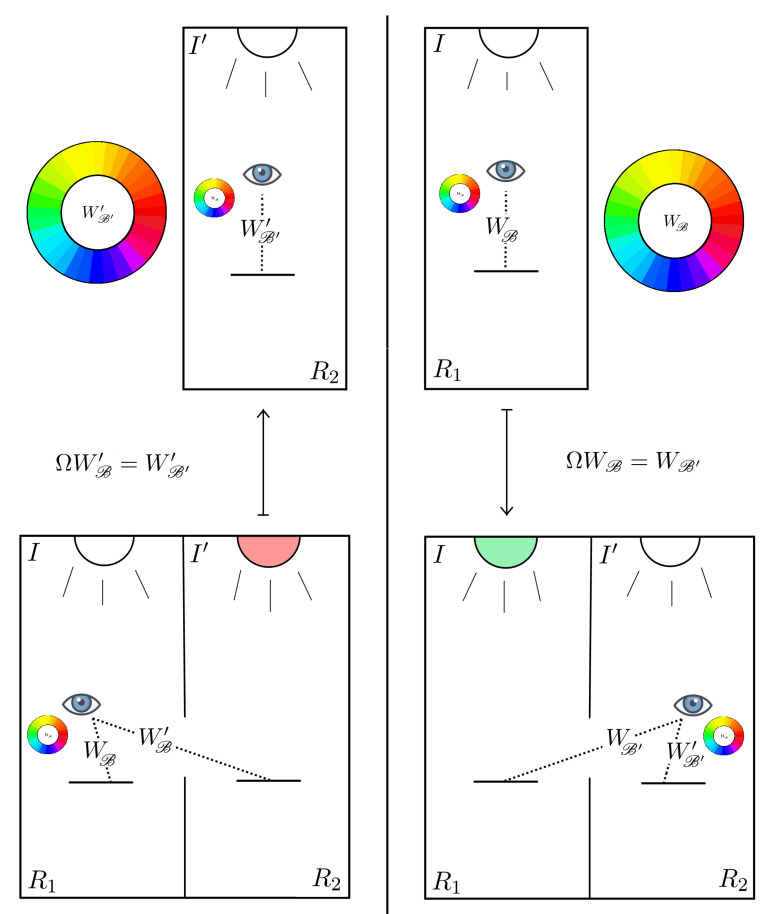
Depiction of Yilmaz’s first experiment with the notation established in Section 3.

Yilmaz’s first experiment contains information about the coordinate change associated to the stimuli *W* and $W^{\prime }$, i.e., $\Omega W_{B}=W_{B^{\prime }}$ and $\Omega W_{B}^{\prime }=W_{B^{\prime }}^{\prime }$, as depicted in Figure 5.

Our aim is to write the coordinates of the four points $W_{B}$, $W_{B^{\prime }}$, $W_{B}^{\prime }$ and $W_{B^{\prime }}^{\prime }$ to determine constraints among the coefficients $\Omega_{ij}$.

An observer adapted to *I* or $I^{\prime }$, respectively, perceives the piece of white paper placed under the same illuminant as the same white. In terms of coordinates, this means that $W_{B}=W_{B^{\prime }}^{\prime }$.

Since the white is achromatic, it must belong to the γ-axis, so its α and β coordinates are null. The third coordinate remains free and we can arbitrarily normalize its value to 1 for simplicity, hence $W_{B}=W_{B^{\prime }}^{\prime }={\left(0,0,1\right)}^{t}$.

Let us now look for the coordinates of $W_{B^{\prime }}=\left(\alpha^{\prime },\beta^{\prime },\gamma^{\prime }\right)$ and $W_{B}^{\prime }=\left(\alpha ,\beta ,\gamma \right)$. As indicated by the notation, $W_{B^{\prime }}$ represents the color sensation of an observer adapted to $I^{\prime }$ when he/she looks at the piece of white paper illuminated by *I* and compares it with the Munsell chips illuminated by $I^{\prime }$. The description of $W_{B}^{\prime }$ is analogous, with *I* and $I^{\prime }$ switched. As reported in Section 2.2, $W_{B^{\prime }}$ is perceived as greenish, i.e., with hue ϕ=π and saturation σ, while $W_{B}^{\prime }$ is perceived as reddish, i.e., with ϕ=0 and saturation σ.

The γ-coordinates $W_{B^{\prime }}$ and $W_{B}^{\prime }$ are not reported by Yilmaz, thus we are led to introduce two unknown parameters $\Gamma ,\tilde{\Gamma }\in R^{+}$ such that $\gamma^{\prime }=\Gamma$ and $\gamma =\tilde{\Gamma }$. Note that Γ and $\tilde{\Gamma }$ are just auxiliary parameters that merely appear in theses intermediate computations and not in the final form of the matrix coefficients of Ω.

The psycho-visual color matching experiments performed by Burnham et al., in the paper [6] imply that the two parameters Γ and $\tilde{\Gamma }$ are actually different from 1. The test results reported in [6] led to the determination of matrices that permit, once the XYZ coordinates of a light patch (i.e., a source of light directly emitting a spectrum) perceived by an observer adapted to *I* are known, to predict the XYZ coordinates of a different light patch having the same appearance for an observer adapted to $I^{\prime }$. In particular, experimental data showed that a patch perceived with the same appearance of white by an observer adapted to the standard CIE illuminants *C* and *A*, i.e., $W_{B}=W_{B^{\prime }}^{\prime }$, has different colorimetric specifications, thus $\gamma_{W_{B}}\neq \gamma_{W_{B}^{\prime }}$. Hence, if we normalize $\gamma_{W_{B}}$ to 1, the value of $\gamma_{W_{B}^{\prime }}=\Gamma$ (and vice versa $\gamma_{W_{B^{\prime }}}=\tilde{\Gamma }$) must be different than 1.

Since the perceived hue of $W_{B^{\prime }}$ is greenish, it must lie on the $\alpha^{\prime }$-axis, i.e., $\beta^{\prime }=0$, thence $\rho^{\prime }=\sqrt{{\alpha^{\prime }}^{2}+{\beta^{\prime }}^{2}}=\left|\alpha^{\prime }\right|$. By definition, $\sigma^{\prime }=\rho^{\prime }/\gamma^{\prime }=\left|\alpha^{\prime }\right|/\Gamma$, but, as reported by Yilmaz, $\sigma^{\prime }=\sigma$, which gives $|\alpha^{\prime }|=\sigma \Gamma$. Finally, since greenish hues lies in the negative part of the $\alpha^{\prime }$-axis, the correct way to write $\alpha^{\prime }$ is as follows: $\alpha^{\prime }=-\sigma \Gamma$. So, $W_{B^{\prime }}={\left(-\sigma \Gamma ,0,\Gamma \right)}^{t}$.

Analogously, we obtain $W_{B}^{\prime }={\left(\sigma \tilde{\Gamma },0,\tilde{\Gamma }\right)}^{t}$, where the positive sign of $\sigma \tilde{\Gamma }$ is due to the fact that, this time, $W_{B}^{\prime }$ is perceived as reddish.

We can now write explicitly the systems $\Omega W_{B}=W_{B^{\prime }}$ and $\Omega^{-1}W_{B^{\prime }}^{\prime }=W_{B}^{\prime }$, by obtaining, respectively:

(6) $$\left(\begin{array}{ccc} \Omega_{11} & 0 & \Omega_{13}\\ 0 & 1 & 0\\ \Omega_{31} & 0 & \Omega_{33} \end{array}\right)\left(\begin{array}{c} 0\\ 0\\ 1 \end{array}\right)=\left(\begin{array}{c} -\sigma \Gamma \\ 0\\ \Gamma \end{array}\right)\;\Leftrightarrow \;\left\{\begin{array}{c} \Omega_{13}=-\sigma \Omega_{33}\\ \Omega_{33}=\Gamma \end{array}\right.\;,$$

(7) $$\left(\begin{array}{ccc} \Omega_{11} & 0 & -\sigma \Omega_{33}\\ 0 & 1 & 0\\ \Omega_{31} & 0 & \Omega_{33} \end{array}\right)\left(\begin{array}{c} \sigma \tilde{\Gamma }\\ 0\\ \tilde{\Gamma } \end{array}\right)=\left(\begin{array}{c} 0\\ 0\\ 1 \end{array}\right)\;\Leftrightarrow \;\left\{\begin{array}{c} \Omega_{11}=\Omega_{33}\\ \Omega_{31}=\frac{1}{\sigma }\left(\frac{1}{\tilde{\Gamma }}-\Omega_{33}\right) \end{array}\right..$$

The only relevant information to retain from the previous equations, in order to determine Ω, is given by the formulae $\Omega_{11}=\Omega_{33}=\Gamma$, $\Omega_{13}=-\sigma \Omega_{11}$, which allow us to write Ω as follows:

(8) $$\Omega =\left(\begin{array}{ccc} \Omega_{11} & 0 & -\sigma \Omega_{11}\\ 0 & 1 & 0\\ \Omega_{31} & 0 & \Omega_{11} \end{array}\right).$$

To determine the remaining parameters we will use the results of the second and the third experiment.

### 3.2. Coefficients from the Second Experiment: The Red Point Transformation

**Figure 6:**
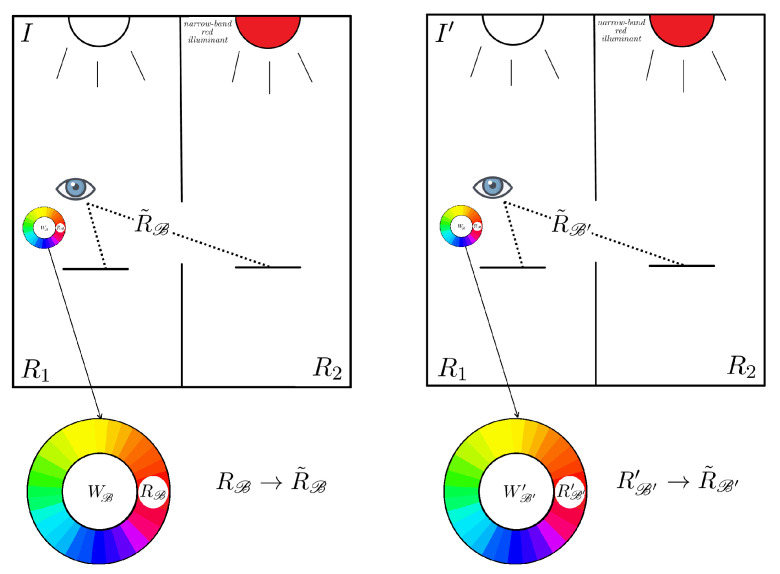
Depiction of Yilmaz’s second experiment with the notation established in Section 3.

Our aim here is to determine the coordinates of ${\tilde{R}}_{B}$ and ${\tilde{R}}_{B^{\prime }}$. Let us denote with $R_{B}$ and $R_{B^{\prime }}^{\prime }$ the maximally saturated Munsell chips with a hue matching that of ${\tilde{R}}_{B}$ and ${\tilde{R}}_{B^{\prime }}$, respectively. The perceived saturation of $R_{B}$ and $R_{B^{\prime }}^{\prime }$ is strictly inferior than $\Sigma_{R}$, see the depiction in Figure 6.

In our mathematical framework, a perceived color is a sensation that can be described in terms of coordinates which come from a match with a set of Munsell chips. The coordinates of ${\tilde{R}}_{B}$ and ${\tilde{R}}_{B^{\prime }}$ will surely depend on $\Sigma_{R}$, which cannot be quantified in the Yilmaz’s setting, thus ${\tilde{R}}_{B}$ and ${\tilde{R}}_{B^{\prime }}$ do not belong to C, but to its boundary ∂C.

The reason why we consider ${\tilde{R}}_{B}$ and ${\tilde{R}}_{B^{\prime }}$ on the boundary of C and not inside C is that we can imagine ${\tilde{R}}_{B}$ and ${\tilde{R}}_{B^{\prime }}$ as resulting from a limit procedure in which a sequence of Munsell chips with increasing saturation approaches their saturation.

The β-coordinate of both ${\tilde{R}}_{B}$ and ${\tilde{R}}_{B^{\prime }}$ is surely 0 because they lie on the α axis. Moreover, their α and γ-coordinates will be $\Sigma_{R}\gamma$ and γ, for $R_{B}$, and $\Sigma_{R}\gamma^{\prime }$ and $\gamma^{\prime }$, with $R_{B}$, $\gamma ,\gamma^{\prime }\in R^{+}$. The unknown parameters γ and $\gamma^{\prime }$ are introduced exactly for the same reason as Γ and $\tilde{\Gamma }$, i.e., we do not know their lightness. As a consequence, ${\tilde{R}}_{B}={\left(\Sigma_{R}\gamma ,0,\gamma \right)}^{t}$ and ${\tilde{R}}_{B^{\prime }}={\left(\Sigma_{R}\gamma^{\prime },0,\gamma^{\prime }\right)}^{t}$.

The equation $\Omega {\tilde{R}}_{B}={\tilde{R}}_{B^{\prime }}$ can be written explicitly as follows:

(9) $$\left(\begin{array}{ccc} \Omega_{11} & 0 & -\sigma \Omega_{11}\\ 0 & 1 & 0\\ \Omega_{31} & 0 & \Omega_{11} \end{array}\right)\left(\begin{array}{c} \Sigma_{R}\gamma \\ 0\\ \gamma \end{array}\right)=\left(\begin{array}{c} \Sigma_{R}\gamma^{\prime }\\ 0\\ \gamma^{\prime } \end{array}\right)\;\Leftrightarrow \;\Omega_{31}=-\frac{\sigma }{\Sigma_{R}^{2}}\Omega_{11},$$

which implies

(10) $$\Omega =\left(\begin{array}{ccc} \Omega_{11} & 0 & -\sigma \Omega_{11}\\ 0 & 1 & 0\\ -\frac{\sigma }{\Sigma_{R}^{2}}\Omega_{11} & 0 & \Omega_{11} \end{array}\right).$$

The explicit form of $\Omega_{11}$ will be obtained thanks to the data gathered from the third experiment.

### 3.3. Coefficients from the Third Experiment: The Yellow Point Transformation

**Figure 7:**
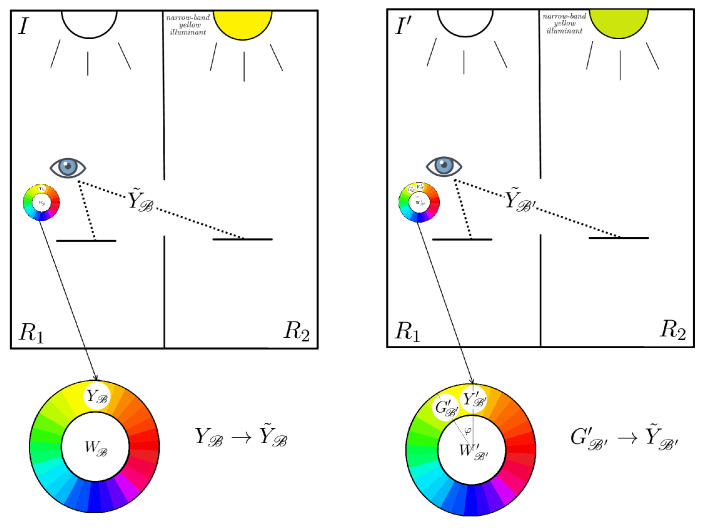
Depiction of Yilmaz’s third experiment with the notation established in Section 3.

When interpreting the third experiment, we will use the same approach as for the second one. We will denote with $Y_{B}$ the maximally saturated Munsell chip with a hue matching that of ${\tilde{Y}}_{B}$. Differently than the second experiment, here, when an observer changes the adaptation state from *I* to $I^{\prime }$, the perceived hue of the narrow band stimulus changes from yellow to a greenish yellow, see Figure 7. For this reason, we denote with $G_{B^{\prime }}^{\prime }$ the maximally saturated Munsell chip that best approximates ${\tilde{Y}}_{B^{\prime }}$.

By using the same arguments of the previous subsections, we write the coordinates of ${\tilde{Y}}_{B}$ as follows: ${\tilde{Y}}_{B}={\left(0,\Sigma_{Y}\tilde{\gamma },\tilde{\gamma }\right)}^{t}$, $\tilde{\gamma }\in R^{+}$. Since the hue of ${\tilde{Y}}_{B^{\prime }}$ increased by an angle φ which satisfies (2), the coordinates of ${\tilde{Y}}_{B^{\prime }}^{\prime }$ are ${\tilde{Y}}_{B^{\prime }}={\left(-\sin\varphi \Sigma_{Y}{\tilde{\gamma }}^{\prime },\cos\varphi \Sigma_{Y}{\tilde{\gamma }}^{\prime },{\tilde{\gamma }}^{\prime }\right)}^{t}$, ${\tilde{\gamma }}^{\prime }\in R^{+}$, where the presence of −sinφ and cosφ comes from the expression of the hue change in Cartesian coordinates.

The equation $\Omega {\tilde{Y}}_{B}={\tilde{Y}}_{B^{\prime }}$ can be written explicitly as follows:

(11) $$\left(\begin{array}{ccc} \Omega_{11} & 0 & -\sigma \Omega_{11}\\ 0 & 1 & 0\\ -\frac{\sigma }{\Sigma_{R}^{2}}\Omega_{11} & 0 & \Omega_{11} \end{array}\right)\left(\begin{array}{c} 0\\ \Sigma_{Y}\tilde{\gamma }\\ \tilde{\gamma } \end{array}\right)=\left(\begin{array}{c} -\sin\varphi \Sigma_{Y}{\tilde{\gamma }}^{\prime }\\ \cos\varphi \Sigma_{Y}{\tilde{\gamma }}^{\prime }\\ {\tilde{\gamma }}^{\prime } \end{array}\right).$$

By direct computation and thanks to Yilmaz’s data $\sin\varphi =\sigma /\Sigma_{Y}$ (this point will be crucial for the following Section 3.4), we obtain

(12) $$\Gamma =\Omega_{11}=\frac{1}{\cos\varphi }=\frac{1}{\sqrt{1-{\left(\frac{\sigma }{\Sigma_{Y}}\right)}^{2}}}.$$

So, at the end, the explicit expression of Ω is:

(13) $$\Omega =\left(\begin{array}{ccc} \Gamma & 0 & -\sigma \Gamma \\ 0 & 1 & 0\\ -\frac{\sigma }{\Sigma_{R}^{2}}\Gamma & 0 & \Gamma \end{array}\right),$$

with Γ as in Equation (12).

The variation of the yellow hue effect is said to be “*similar to the aberration effect in special relativity*”, by Yilmaz in [1] at page 132. We will discuss this aspect in Section 5.

In Section 4, we will point out the analogy between Ω and the matrix that represents Lorentz’s transformations in Einstein’s theory of special relativity.

### 3.4. Critical Issues in Yilmaz’s Model

In the previous subsections our aim was to recast Yilmaz’s model in a rigorous framework, with respect to both its colorimetric interpretation and its mathematical development, remaining as close as possible to what Yilmaz reported.

In this subsection, instead, we point out some critical issues about Yilmaz’s model that are essential to underline before carrying on our analysis about similarities and differences between Yilmaz’s results and special relativity.

The first issue to discuss is the incongruous use of Munsell chips in its experiments. The piece of white paper is used merely as a sort of ideal non-selective Lambertian reflector for the illuminant, thus, comparing the color sensation induced by it with a set of Munsell chips is not coherent. A clear, fundamental, manifestation of this lack of coherence is the fact that no Munsell chip can be found to match the saturation of a narrow band illuminant reflected by the piece of white paper.

While the set of Munsell chips was an obvious choice in 1962, the year of publishing of Yilmaz’s paper, nowadays we can replace it without effort with an emitting display that will also allow us performing comparisons with narrow-band lights. With such a modern experimental apparatus, the color sensations ${\tilde{R}}_{B},{\tilde{R}}_{B^{\prime }},{\tilde{Y}}_{B}$ and ${\tilde{Y}}_{B^{\prime }}$ will be effectively measurable and thus be considered as perceived colors. Moreover, this emitting display will also have the advantage of not be affected by the presence of the illuminant.

Another questionable issue in Yilmaz’s paper is the fact that he does not exclude the dependency of the maximal saturation Σ on the hue ϕ. The saturation of a color sensation is defined as a percentage: 100% representing the absence of a washed-out sensation, as it happens for a narrow-band light, and 0% corresponding to the totally washed-out sensation of achromatic stimuli. These measurements are independent on the hue ϕ, so it does not make sense to assume that the maximal saturation Σ depends on ϕ.

From now on, we will remove the subscript ϕ from Σ and consider it as a constant. With this choice, Equations (12) and (13) become, respectively:

(14) $$\Gamma =\frac{1}{\sqrt{1-{\left(\frac{\sigma }{\Sigma }\right)}^{2}}},$$

and

(15) $$\Omega =\left(\begin{array}{ccc} \Gamma & 0 & -\sigma \Gamma \\ 0 & 1 & 0\\ -\frac{\sigma }{\Sigma^{2}}\Gamma & 0 & \Gamma \end{array}\right).$$

Finally, it still remains unclear if the results claimed by Yilmaz have been obtained after actual observations or if they are the results of a *gedankenexperiment*, i.e., a thought experiment. In the first case, Yilmaz does not report any experimental data and they do not seem to be found anywhere else, this, of course, raises more than a doubt about their validity.

In the second case, it is clear that Yilmaz pushed the gedankenexperiment technique way too far: a thought experiment is used to check what known results of a given theory would predict in an experimental configuration that is not possible to test with the current available technology. No known colorimetric result can be used to predict the outcomes of the three experiments, in particular, we notice that the hue shift in the third experiment represented by Equation (2) seems to be somehow forced to have that analytical expression to adjust the equations that permit to determine the matrix Ω with the desired expression of its coefficients.

In spite of the critical issues just underlined, Yilmaz’s paper has the great merit of highlighting the theoretical importance of the assumption that the maximal saturation Σ of the color perceived from spectral lights is invariant w.r.t. changes of illuminants.

## 4. Yilmaz’s Model and the Standard Formulation of Special Relativity

In this section, we are going to show that it is possible to obtain the same results as Yilmaz’s ones by replacing his controversial experimental results with assumptions on the geometric structure of the perceived color space C. It is important to stress that our aim here is purely theoretical: we are interested in showing to what extent Yilmaz’s model is related to the classical formulation of special relativity and what constraints C must satisfy in order to maximize the analogies between the two theories. These constraints, as we will see, are far from being mild and this is the reason why we consider alternative formulations of special relativity, such as, e.g., those that originated from Mermin’s paper [7], which are more appropriate to formulate a relativistic theory of color perception. We discuss this issue in the discussion section.

### 4.1. Elements of Special Relativity

Here we will briefly recap only the basic concepts of special relativity that are needed to show analogies and differences with Yilmaz’s model. The discussion that follows will be faithful to the standard special relativity formulation, see, e.g., [8,9].

Special relativity is known to be an extension of Galilean relativity, which is based on the following two postulates:

1. space is homogeneous and isotropic and time is homogeneous (in this context, isotropy means invariance under rotations, while homogeneity means invariance with respect to translations);
2. laws of physics have the same form in all inertial (i.e., not accelerated) reference frames, i.e., no inertial reference frame is privileged.

In special relativity, Einstein considered, along with the motion of objects with mass, also the peculiar behavior of electromagnetic signals by adding the following postulate:

3. the speed of light in vacuum has a constant value $c\in R^{+}$ when measured in all inertial reference frames.

In special relativity, we call *event* *e* a point in $R^{4}=R\times R^{3}$ with coordinates written as $x^{\mu }=\left(ct,x\right)$, where t∈R and $x=(x^{i})$, i=1,2,3, are, respectively, the time instant and the spatial position of the event as measured by an inertial observer with respect to her/his inertial reference frame R. Using ct instead of *t* is customary in special relativity: physically, this amounts at replacing the time *t* with the corresponding space ct traveled by a ray of light during *t*. Let us consider, in particular, the following two events: the first, $e_{1}=\left(ct_{1},x_{1}^{i}\right)$, consists in a light signal emanating at the time $t_{1}$ from the spatial position $\left(x_{1}^{i}\right)$; the second, $e_{2}=\left(ct_{2},x_{2}^{i}\right)$, consists in the same light signal arriving at the time $t_{2}$ in the spatial position $\left(x_{2}^{i}\right)$. Since the signal propagates with constant speed *c*, the square distance that is traveled is $c^{2}{\left(t_{2}-t_{1}\right)}^{2}$. If we equip $R^{3}$ with the Euclidean metric, this same square distance is equal to $\sum_{i=1}^{3}{\left(x_{2}^{i}-x_{1}^{i}\right)}^{2}$, so the coordinates of the events $e_{1}$ and $e_{2}$ in the fixed inertial frame R are related by the equation:

(16) $$c^{2}{\left(t_{2}-t_{1}\right)}^{2}-\sum_{i=1}^{3}{\left(x_{2}^{i}-x_{1}^{i}\right)}^{2}=0\Leftrightarrow c^{2}{\left(t_{2}-t_{1}\right)}^{2}-{∥x_{2}-x_{1}∥}^{2}=0,$$

∥·∥ being the Euclidean norm in $R^{3}$. Of course, Equation (16) remains valid for all spacetime differences, also infinitesimal ones, thus we can write the differential version of Equation (16) as $c^{2}dt^{2}-{∥dx∥}^{2}=0$. In special relativity, the quantity

(17) $$ds^{2}=c^{2}dt^{2}-{∥dx∥}^{2},$$

is called *spacetime interval*. From Equation (16) it follows that the spacetime interval between two events connected by a signal traveling at the speed of light is null. Since the speed of light is an upper limit for velocity, this amounts at promoting it as a reference and at normalizing to 0 the spacetime distance between any two events, no matter how far in space or time, connected by a light-speed signal.

Postulates 1 and 3 imply that the spacetime interval $ds^{2}$ between two events described in the inertial reference frame R and the spacetime interval $ds^{\prime 2}$ between the same couple of events described in any other inertial reference frame $R^{\prime }$ is exactly the same: $ds^{\prime 2}=ds^{2}$, see, e.g., [9], page 7 or [8], page 117, for a rigorous proof.

We will make use of the standard **Einstein’s convention** which implicitly assumes a sum over repeated indices *above and below* in an algebraic expression, the sum being of course performed over the range of index variability, e.g., if i=1,⋯,n, then $a^{i}b_{i}:=\sum_{i=1}^{n}a_{i}b_{i}.$ This notation is consistent as long as we agree to write the indices below for the basis vectors and above for the components w.r.t. them. If we write the infinitesimal difference between any two events as the vector $dx=(dx^{\mu })$, then the spacetime interval can be written as the (non positive-definite) quadratic form $ds^{2}=dx^{\mu }\eta_{\mu \nu }dx^{\nu }=dx^{t}\eta dx$, where $\eta =(\eta_{\mu \nu })$ is the matrix η= diag (1,−1,−1,−1). The metric space $M=(R^{4},\eta )$ is called *Minkowski spacetime* and η is the matrix associated to the Minkowski quadratic form. The associated pseudo-norm, i.e., ${∥u∥}_{M}^{2}={\left(u^{0}\right)}^{2}-\left[{\left(u^{1}\right)}^{2}+{\left(u^{2}\right)}^{2}+{\left(u^{3}\right)}^{2}\right]$ is called *Minkowski norm* of u∈M.

A *world-line* in M is any connected path composed by events between an initial and a final one. Straight lines in M correspond to world-lines of inertial movements.

The last information that we must recall is how to relate the coordinates of two inertial frames. First of all, it is simple to deduce from postulate 1 that the coordinate transformation $\omega :R^{4}\to R^{4}$, $x^{\mu }\mapsto x^{\prime \mu }=\omega \left(x^{\mu }\right)$ from R to $R^{\prime }$ of an event must be *linear* (under the reasonable hypothesis to be differentiable). In fact, by postulate 1, there are no special instants and positions in $R^{4}$, so, the distance between two events remains the same when these are translated by a fixed vector $b\in R^{4}$. This is true independently on the coordinate system used to write the events in two arbitrary inertial reference frames R and $R^{\prime }$. Let $x=x^{\mu }$ and $y=y^{\mu }$ be the coordinates of the two events in R and $\omega^{\mu }\left(x\right)$ and $\omega^{\mu }\left(y\right)$ the coordinates of the same events in $R^{\prime }$. Since $\left(x^{\mu }+b^{\mu }\right)-\left(y^{\mu }+b^{\mu }\right)=x^{\mu }-y^{\mu }$, we must have $\omega^{\mu }\left(x+b\right)-\omega^{\mu }\left(y+b\right)=\omega^{\mu }\left(x\right)-\omega^{\mu }\left(y\right)$. If we derive the two sides of the last equation with respect to $x^{\nu }$, ν=0,1,2,3, we obtain $\frac{\partial \omega^{\mu }}{\partial x^{\nu }}\left(x+b\right)=\frac{\partial \omega^{\mu }}{\partial x^{\nu }}\left(x\right)$, for all $b\in R^{4}$, since *y* does not depend on *x*. Thanks to the fact that *b* is arbitrary, x+b represents any vector in $R^{4}$, so the function $\frac{\partial \omega^{\mu }}{\partial x^{\nu }}$ is constant, which implies that $\frac{\partial \omega^{\mu }}{\partial x^{\nu }}\left(x\right)=\Lambda_{\;\nu }^{\mu }\in R$ for all $x\in R^{4}$, μ,ν=0,1,2,3, i.e.,

(18) $$x^{\prime \mu }=\omega^{\mu }\left(x\right)=\Lambda_{\;\nu }^{\mu }x^{\nu }+a^{\mu }.$$

The invariance of the spacetime interval imposes a strong constraint on the form of the matrix Λ: to see this, let us write the difference vector $dx^{\mu }$ in the inertial reference frame $R^{\prime }$ by using Equation (18): $dx^{\prime \mu }=y^{\prime \mu }-x^{\prime \mu }=\Lambda_{\;\nu }^{\mu }y^{\nu }+a^{\mu }-\left(\Lambda_{\;\nu }^{\mu }x^{\nu }+a^{\mu }\right)=\Lambda_{\;\nu }^{\mu }dx^{\nu }$. Thus, on one side,

(19) $$ds^{\prime 2}=dx^{\prime \mu }\eta_{\mu \nu }dy^{\prime \nu }=dx^{\alpha }\Lambda_{\;\alpha }^{\mu }\eta_{\mu \nu }\Lambda_{\;\beta }^{\nu }dy^{\beta },$$

and, on the other side,

(20) $$ds^{2}=dx^{\alpha }\eta_{\alpha \beta }dy^{\beta },$$

so, the equality $ds^{\prime 2}=ds^{2}$ implies:

(21) $$\Lambda_{\;\alpha }^{\mu }\eta_{\mu \nu }\Lambda_{\;\beta }^{\nu }=\eta_{\alpha \beta }\;\Leftrightarrow \;\Lambda^{t}\eta \Lambda =\eta .$$

The set of all these matrices forms a group, called the *Lorentz group* classically denoted by the symbols $L\equiv O\left(1,3\right)=\{\Lambda \in GL\left(4,R\right)\;:\;\Lambda^{t}\eta \Lambda =\eta \}$.

Thus, postulates 1 and 3 imply that the coordinates used to describe the same event in two generic inertial reference frames are related by either non-homogeneous linear transformations of the type $x^{\prime }=\Lambda x+a$, Λ∈O(1,3), $a\in R^{4}$, called *Poincaré transformations*, or, in the special case when a=0, by linear transformations

(22) $$x^{\prime }=\Lambda x,$$

called *Lorentz transformations*.

### 4.2. Similarities and Differences between Yilmaz’s Model and Special Relativity

Table 1 provides the list of analogies between Yilmaz’s model and the standard mathematical framework of special relativity just recalled.

Notice that, if homogeneity and isotropy of C are assumed, then the linear nature of Yilmaz transformations is not only approximated, but exact, as previously pointed out in Section 4.1.

Among the similarities listed above, substantial differences between special relativity and Yilmaz’s model of color perception must be remarked.

1. The Helson-Judd effect, see, e.g., [10], shows that human color perception experiences an *incomplete adaptation* to narrow-band illuminants, thus, in the previous table, the analogy between inertial frames and observers works only if they are adapted to broadband illuminants.
2. While time *t* can be extended to the whole R with the identification of negative values of *t* as the ‘past’, a negative lightness is meaningless. So, only the upper part of the cone C makes sense in color perception. Moreover, and most importantly, this cone is not infinite: in fact, it is bounded from above by the glare limit defined by $\gamma_{\max}$ and from below for two reasons: the first is the Purkinje effect [11] when we pass from photopic to scotopic vision via the mesopic range (in the photopic range the three retinal cones are activated, in the scotopic range only the retinal rods are, while in the mesopic both photoreceptors function simultaneously), and the second is the intensity threshold of the retinal rods. Thus, C is a *truncated cone* defined by the equation $C=\{F=\left(\phi ,\rho ,\gamma \right)\in \left[0,2\pi \right)\times R^{+}\times \left[\gamma_{\min},\gamma_{\max}\right]\;|\;\rho <\Sigma \gamma \}$.
3. While events in the Minkowski spacetime have four components, perceived colors have only three.

The similarities above lead naturally to the question if it is possible to endow C with a Minkowski-like metric and, if so, under what set of assumptions about color perception. We investigate this issue in the following subsection.

### 4.3. The Issue of a Minkowski-Like Metric on C

Homogeneity and isotropy of spacetime, the absence of a preferred inertial observer and the constancy of light speed in vacuum for inertial frames naturally leads to endow $R^{4}$ with the Minkowski metric. Similarly, in Yilmaz’s model, the perceived colors space is homogeneous and isotropic (no combination of hue, saturation and intensity is ‘special’ as long as we remain in C), no observer adapted to a broadband illuminant can be considered privileged, and the maximal saturation Σ is invariant under changes of broadband illuminants.

It is thus tempting to check if it is possible to repeat, in the case of Yilmaz’s color perception model, the same argument used in special relativity to single out the Minkowski metric, which, at page 26 of [1], is claimed to induce a geometry on the color space which leads to “*a good approximation of color vision phenomena*”. As we will see, this is possible only under the hypothesis that the hue-chroma plane is endowed with the Euclidean metric.

Let us start this investigation by considering two spectral colors $F_{1}$ and $F_{2}$ belonging to ∂C with the same hue ϕ, but with different values of γ. Let us fix the axis $\hat{\alpha }$ in the direction defined by ϕ, so that the coordinates of $F_{1}$ and $F_{2}$ are: $F_{1}=\left(\alpha_{1},0,\gamma_{1}\right)$ and $F_{2}=\left(\alpha_{2},0,\gamma_{2}\right)$. Since $F_{1}$ and $F_{2}$ are spectral colors, by definition they have maximal saturation Σ, so that we can write the system

$$\left\{\begin{array}{c} \Sigma \gamma_{1}=\alpha_{1}\\ \Sigma \gamma_{2}=\alpha_{2} \end{array}\right.,$$

by subtracting the first equation from the second we obtain $\Sigma^{2}{\left(\gamma_{2}-\gamma_{1}\right)}^{2}={\left(\alpha_{2}-\alpha_{1}\right)}^{2}$, whose infinitesimal version is $\Sigma^{2}d\gamma^{2}=d\alpha^{2}$.

This result tells us that, if we endow *the intersection between C and the plane defined by any fixed value of ϕ* with the metric

(23) $$ds^{2}=\Sigma^{2}d\gamma^{2}-d\alpha^{2},$$

then we consider as having 0 perceptual distance any two points in C with the same hue and maximal saturation, in spite of having different chroma and lightness, when described by two observers adapted to different illuminants. Thus, the distance (23) comes from promoting spectral lights, having the maximal attainable saturation, to a reference and normalizing their distance to 0.

Without specific hypotheses, there are infinite ways to extend the metric $\phi \mapsto ds_{\phi }^{2}=\Sigma^{2}d\gamma^{2}-d\rho^{2}$, defined for all *fixed* ϕ∈[0,2π), on the whole C. In fact, every metric of the type

(24) $$ds^{2}=fd\phi^{2}+gd\gamma d\phi +hd\rho d\phi -d\rho^{2}+\Sigma^{2}d\gamma^{2},$$

where f,g,h are are arbitrary functions of ϕ,ρ,γ, i=1,2,3, is an extension of (23) one because, when (24) is restricted to the plane defined by ϕ= constant, the differential of ϕ vanishes and all the terms containing dϕ disappear.

In an orthogonal coordinate system (w.r.t., the Euclidean inner product), the metric (24) becomes diagonal, so that we can write

(25) $$ds^{2}=f\left(\phi ,\rho ,\gamma \right)d\phi^{2}-d\rho^{2}+\Sigma^{2}d\gamma^{2}.$$

We are going to discuss the assumptions that permit to single out the specific analytical form of *f*. The first assumption seems the most reasonable one: if we make the hypothesis that no hue can be considered as special, then *f* does not depend on ϕ and (25) becomes:

(26) $$ds^{2}=f\left(\rho ,\gamma \right)d\phi^{2}-d\rho^{2}+\Sigma^{2}d\gamma^{2}\equiv ds_{c}^{2}+\Sigma^{2}d\gamma^{2},$$

where $ds_{c}^{2}=f\left(\rho ,\gamma \right)d\phi^{2}-d\rho^{2}$ is the metric $ds^{2}$ restricted to the chromaticity plane by fixing γ.

Let us now introduce *the strongest hypothesis* of our extension procedure: we assume that the chromaticity plane is endowed with the Euclidean metric. This is the standard choice that is made in classical colorimetry (actually, in classical CIE colorimetry, the whole color space is endowed with a Euclidean color metric), see, e.g., [12] and the references therein, however, mathematically speaking, *this choice is completely arbitrary*. $ds_{c}^{2}$ is coherent with a Euclidean metric in polar coordinates on the chromaticity plane, i.e., $ds_{E}^{2}=\rho^{2}d\phi^{2}+d\rho^{2}$, if and only if *f* has the following separate dependence on ρ and γ: $f\left(\rho ,\gamma \right)=-\rho^{2}\tilde{f}\left(\gamma \right)$, where $\tilde{f}$ is an arbitrary positive-valued function of γ, where positivity must be requested to preserve the signature of the Euclidean metric. The term ‘coherent’ here is used in the sense that, for every fixed value of $\gamma =\bar{\gamma }$, the metric restricted to the plane $\gamma =\bar{\gamma }$ is the Euclidean metric in polar coordinates up to a constant. The constant is $\tilde{f}\left(\bar{\gamma }\right)$ and, since it is positive, we can rescale the variable $\phi \mapsto \phi /\sqrt{\tilde{f}\left(\bar{\gamma }\right)}$ in order to obtain exactly the Euclidean metric on the plane. To resume, the hypothesis that the chromaticity plane is equipped with a Euclidean metric implies that (26) must have the following expression:

(27) $$ds^{2}=-\rho^{2}\tilde{f}\left(\gamma \right)d\phi^{2}-d\rho^{2}+\Sigma^{2}d\gamma^{2}.$$

The only degree of freedom that remains left is the function $\tilde{f}$, however, we are going to show that, if one also assumes the homogeneity hypothesis of C under scaling of γ and ρ, then $\tilde{f}$ must be constant. In fact, if we transform the color coordinates with a uniform scaling, i.e., we perform (ϕ,ρ,γ)↦(ϕ,λρ,λγ), λ>0, then, homogeneity implies that the metric must change as follows: $ds^{2}\mapsto \lambda^{2}ds^{2}$. This implies that $\tilde{f}\left(\lambda \gamma \right)=\tilde{f}\left(\gamma \right)$ for all λ>0, i.e., that $\tilde{f}\left(\gamma \right)=k$, k>0 constant, for all γ.

If we rescale the hue ϕ as follows $\phi \mapsto \phi /\sqrt{k}$, then (27) becomes

(28) $$ds^{2}=-\rho^{2}d\phi^{2}-d\rho^{2}+\Sigma^{2}d\gamma^{2},$$

which is the expression of the Minkowski metric on C in polar coordinates.

To resume, the classical hypothesis on the geometry of the space of perceived colors C, i.e., homogeneity w.r.t ρ and γ, isotropy w.r.t ϕ and the choice to measure distances on the hue-chroma plane by means of a Euclidean metric, single out the Minkowski metric on C as the only one compatible with these classical colorimetric hypothesis.

If Yilmaz’s assumptions about the perceptual effect of (broadband) illuminant changes being described by Lorentz transformations and about the constancy of Σ are accepted, then the same arguments used in the standard formalism of special relativity, and quoted in Section 4.1, can be used to check that the Minkowski metric on C is invariant under (broadband) illuminant changes.

Finally, we remark that Yilmaz’s transformations, represented by the matrix Ω of Equation (15), are isometries for the Minkowski metric. In fact, if F∈C has coordinates $F={\left(\alpha ,\beta ,\gamma \right)}^{t}$ w.r.t to a basis B, then its Minkowski norm is:

(29) $${∥F∥}^{2}=-\alpha^{2}-\beta^{2}+\Sigma^{2}\gamma^{2},$$

while

(30) $$\Omega F=\left(\begin{array}{ccc} \Gamma & 0 & -\sigma \Gamma \\ 0 & 1 & 0\\ -\frac{\sigma }{\Sigma^{2}}\Gamma & 0 & \Gamma \end{array}\right)\left(\begin{array}{c} \alpha \\ \beta \\ \gamma \end{array}\right)=\left(\begin{array}{c} (\alpha -\sigma \gamma )\Gamma \\ \beta \\ (\gamma -\frac{\sigma }{\Sigma^{2}}\alpha )\Gamma \end{array}\right)$$

has Minkowski norm

(31) $$\begin{array}{cc} {∥\Omega F∥}^{2}= & {\left(\Omega F\right)}^{t}g\left(\Omega F\right)=-{\left(\alpha -\sigma \gamma \right)}^{2}\Gamma^{2}-\beta^{2}+\Sigma^{2}{\left(\gamma -\frac{\sigma }{\Sigma^{2}}\alpha \right)}^{2}\Gamma^{2}\\ = & -\beta^{2}+\Gamma^{2}\left(-\alpha^{2}-\sigma^{2}\gamma^{2}+\gamma^{2}\Sigma^{2}+\frac{\sigma^{2}}{\Sigma^{2}}\alpha^{2}\right)\\ = & -\alpha^{2}-\beta^{2}+\Sigma^{2}\gamma^{2}\\ = & {∥F∥}^{2}, \end{array}$$

i.e., ∥ΩF∥=∥F∥ for all F∈C.

## 5. Relativistic Aberration and Yilmaz’s Third Experiment

In this paragraph, we want to discuss Yilmaz’s most ambiguous assumption, represented by the result of the third experiment, in a relativistic framework and show that it is the translation, in the colorimetric context, of the relativistic aberration effect. This phenomenon expresses how the angle of incidence of a ray of light changes with the inertial frame of reference and it is a direct application of Lorentz transformations.

Let R and $R^{\prime }$ be two inertial reference frames, with $R^{\prime }$ moving with respect to R with constant speed *v* along the *x*-direction. Without loss of generality we can consider a photon moving towards the origin of the frame and whose spatial trajectory is a straight line contained in the plane z=0. Of course, in both R and $R^{\prime }$, the speed of the photon will be *c*.

We suppose that its trajectory forms the angle α (resp. $\alpha^{\prime }$) in R (resp. $R^{\prime }$), with the *x*-direction shared by both R and $R^{\prime }$. Our aim is to show the functional relationship between α and $\alpha^{\prime }$. In R the photon’s world-line is given by (t,x,y,z)=(t,−tccosα,−tcsinα,0), to obtain it with respect to $R^{\prime }$ we need to apply the so-called Lorentz boost as follows:

(32) $$\left\{\begin{array}{c} t^{\prime }=\gamma \left(t-\frac{v}{c^{2}}x\right)\\ x^{\prime }=\gamma \left(x-vt\right)\\ y^{\prime }=y\\ z^{\prime }=z \end{array}\right.,$$

with $\gamma =\frac{1}{\sqrt{1-v^{2}/c^{2}}}$.

In particular $x^{\prime }=\gamma \left(-vt-ct\cos\alpha \right)=-ct^{\prime }\cos\alpha^{\prime }$ and $y^{\prime }=y=-ct\sin\alpha =-ct^{\prime }\sin\alpha^{\prime }$.

Hence

(33) $$\tan\alpha^{\prime }=\frac{y^{\prime }}{x^{\prime }}=\frac{c\sin\alpha }{\gamma (c\cos\alpha +v)}=\frac{\sin\alpha \sqrt{1-v^{2}/c^{2}}}{\cos\alpha +\frac{v}{c}}.$$

By a straightforward calculation we obtain

(34) $$\cos^{2}\alpha^{\prime }=\frac{1}{1+\tan^{2}\alpha^{\prime }}=\frac{{\left(\cos\alpha +\frac{v}{c}\right)}^{2}}{{\left(1+\frac{v}{c}\cos\alpha \right)}^{2}},$$

thus

(35) $$\cos\alpha^{\prime }=\frac{\cos\alpha +\frac{v}{c}}{1+\frac{v}{c}\cos\alpha },$$

where only the positive determination of the square root is compatible with the fact that, if v=0, then we must have $\cos\alpha^{\prime }=\cos\alpha$. Moreover

(36) $$\cos\alpha^{\prime }-\cos\alpha =\frac{\frac{v}{c}\sin^{2}\alpha }{1+\frac{v}{c}\cos\alpha }>0,$$

indeed 0<v<c, so $\cos\alpha^{\prime }>\cos\alpha$ and $\alpha^{\prime }<\alpha$.

We have now all the information to discuss Yilmaz’s third experiment: taking into account the analogies that we have commented in Section 4 together with Equation (35), we have that

(37) $$\cos\phi^{\prime }=\frac{\Sigma \cos\phi +\sigma }{\Sigma +\sigma \cos\phi }.$$

For the spectral yellow, we have that ϕ=π/2, so Equation (Section 5) becomes $\cos\phi^{\prime }=\frac{\sigma }{\Sigma }$, but since $\varphi =\phi -\phi^{\prime }$, we get

(38) $$\sin\varphi =\sin\left(\frac{\pi }{2}-\phi^{\prime }\right)=\cos\phi^{\prime }=\frac{\sigma }{\Sigma }$$

which corresponds to the Equation (2) reported by Yilmaz, concerning the hue variation of the spectral yellow.

## 6. Discussion

Yilmaz’s contribution [1], based on the analogies between color perception and special relativity, stood out from the classical CIE color space theories for at least two reasons: the first is that he was able to model the perceptual effect of illuminant changes via Lorentz transformations and the second is that he put the accent on the possibility to replace the standard Euclidean CIE metrics with a 3D version of the Minkowski metric.

However, as we have shown in Section 4.3, the possibility to endow C with the Minkowski metric coherently with the rest of Yilmaz’s assumptions requires to impose a Euclidean metric on the hue-chroma plane obtained by fixing a constant value for the lightness γ. This underlines a systematic and important difference between Yilmaz’s model and the CIE classical color space models. In fact, while these latter are globally Euclidean spaces, the fact that Yilmaz introduced the Minkowski metric in the space C allows us to investigate naturally hyperbolic structures associated with the Minkowski metric, i.e., hyperbolic leaves that foliate C that coincide with surfaces of constant Minkowski norm. This natural association is not possible in the Euclidean space endowed with the canonical Euclidean norm.

We are currently investigating an alternative formulation of Yilmaz’s model in which hyperbolicity is present also on the chromatic plane: in fact, if we consider it to be the hue-saturation plane instead of the hue-chroma one, then it is possible to endow it with a hyperbolic metric. The reason behind this fact is that, while the hue-chroma plane is simply a slice of the complete color space, the hue-saturation plane is obtained via a hyperbolic projection.

The determination of the formal relationship between these two alternative formulations is still an open problem that clearly underlines the important issue of a coherent understanding and definition of the colorimetric attributes.

This involves also the correct interpretation of the coordinate γ: there are several visual phenomena, e.g., the Helmholtz-Kohlrausch effect, the Bezold-Brücke hue shift and the Hunt Effect [10], which show that treating γ as independent of the chromatic coordinates is not coherent with human perception. A rigorous mathematical model of the relationship between γ and the chromatic coordinates of a perceived color is a subtle open problem that we deem important to solve.

Yilmaz himself, in the last part of his paper, quoted the Bezold-Brücke hue shift as “*a departure from the Minkowski metric*”, this observation led him conjecture that, in order to take into account this effect, C should be a negatively curved color space endowed with a more complicated metric.

Remarkably, these Yilmaz’s speculations had a strong impact on H.L. Resnikoff who, in the paper [13] published twelve years later, acknowledged Yilmaz for his intuition and proved rigorously that a hyperbolic color space, i.e., a homogeneous space with constant negative curvature, is perfectly compatible with the phenomenology of color perception, see also [14] for a modern discussion.

Finally, we would like, in future investigations, to understand how the mathematical properties of the perceived color space relate with the physics of color, which is still a hard open problem to solve.

## Figures and Tables

**Table 1:** Analogies between special relativity and Yilmaz’s model of color perception.

Special Relativity	Yilmaz’s Color Perception Model
Homogeneous and isotropic spacetime	Homogeneous and isotropic color space
Observer in an inertial frame	Observer adapted to a broadband illuminant
Event $e=\left(t,x\right)\in R^{4}$	Perceived color F=(ϕ,ρ,γ)∈C
Time coordinate t∈R	Lightness coordinate $\gamma \in R^{+}$
Spatial coordinates $\left(x^{1},x^{2},x^{3}\right)\in R^{3}$	Chromatic coordinates $\left(\rho ,\phi \right)\in R^{+}\times \left[0,2\pi \right)$
Speed of light in vacuum *c*	Maximal perceived saturation Σ
Lorentz transformations (22)	Yilmaz transformations (13)
